# Neoadjuvant Chemotherapy and Stereotactic Body Radiation Therapy in Patients with Early-Onset Pancreatic Cancer: Clinical Outcomes and Toxicity

**DOI:** 10.3390/cancers18091418

**Published:** 2026-04-29

**Authors:** Shuchi Sehgal, Abhinav V. Reddy, Suqi Ke, Colin S. Hill, Timothy A. Lin, Serena Mao, Lei Zheng, Jin He, Joseph M. Herman, Chen Hu, Jeffrey J. Meyer, Amol K. Narang

**Affiliations:** 1 Department of Radiation Oncology and Molecular Radiation Sciences, Johns Hopkins University School of Medicine, Baltimore, MD 21287, USAanarang2@jhmi.edu (A.K.N.); 2 Northside Radiation Oncology Consultants, Canton, GA 30115, USA; 3 Department of Radiation Oncology, NYU Langone Health, New York, NY 10016, USA; 4 Department of Radiation Oncology, Division of Radiation Oncology, The University of Texas MD Anderson Cancer Center, Houston, TX 77030, USA; 5 George Washington Medical Faculty Associates, Washington, DC 20037, USA; 6 Department of Medical Oncology, Johns Hopkins University School of Medicine, Baltimore, MD 21287, USA; 7 Department of Surgery, Johns Hopkins University School of Medicine, Baltimore, MD 21287, USA; 8 Department of Radiation Oncology, Northwell Health Cancer Institute, New Hyde Park, NY 11042, USA

**Keywords:** pancreatic adenocarcinoma, stereotactic body radiation therapy, early onset

## Abstract

**Purpose/Objective**: This purpose of this study is to report on a cohort of patients with Early-onset pancreatic cancer (EOPC) (age < 55 years) who were treated with neoadjuvant chemotherapy and stereotactic body radiation therapy (SBRT) with or without surgical resection. **Materials/Methods**: This was a single-institution, retrospective review of patients with EOPC that was staged as either borderline resectable (BRPC) or locally advanced (LAPC) based on NCCN criteria and who were treated with upfront chemotherapy followed by SBRT, with or without subsequent surgical resection. Endpoints included overall survival (OS), local progression-free survival (LPFS), distant metastasis-free survival (DMFS), progression-free survival (PFS), and treatment-related toxicity. **Results**: From 2016 to 2021, 47 patients met the inclusion criteria. The median age was 50.4 years (range, 36.4–54.7 years). The median duration of induction chemotherapy was 4 months (range, 2.5–9.0 months). The SBRT dose for the majority (46/47, 97.9%) of patients was 33 Gy in five fractions. Surgical resection was performed in 33 patients (70.2%). The median OS, LPFS, DMFS, and PFS were 14.2 months, 11.6 months, 8.9 months, and 8.1 months respectively. Six-month and 1-year LPFS were 88.3% and 45.4%, respectively. Chemotherapy duration (≥4 months) was associated with improved median OS (16.5 vs. 10.1 months, *p* = 0.005), LPFS (10.1 vs. 4.9 months, *p* = 0.002), DMFS (9.7 vs. 5.2 months, *p* = 0.014), and PFS (9.7 vs. 5.2 months, *p* = 0.020). The normalization of CA 19-9 (≤34 vs. >34 U/mL) after chemotherapy was associated with improved median DMFS (not reached vs. 5.6 months, *p* = 0.003) and PFS (11.3 vs. 5.6 months, *p* = 0.022). Grade 3+ rates of chemotherapy and radiation-related toxicity were 14.9% and 2.1% respectively. The Clavien–Dindo 3b toxicity rate was 3.0%. **Conclusions**: In EOPC, an induction chemotherapy duration ≥4 months and the normalization of CA 19-9 after chemotherapy were associated with improved outcomes, suggesting a role for extended durations of systemic therapy titrated to CA 19-9 response before transitioning to local therapy. The high rate of local failure and the low rate of grade 3+ toxicity also suggest a role for intensifying local therapy in this population.

## Simple Summary

Little is known about Early-onset pancreatic cancer (EOPC) (age < 55 years), since most patients diagnosed with pancreatic cancer are around the age of 70. This article aims to identify a pathway which will help draw clear conclusions with respect to optimal treatment paradigms for EOPC across specific stages of the disease. Thus, we report on the clinical outcomes and toxicity of a cohort of patients with EOPC diagnosed and treated for borderline or locally advanced pancreatic cancer (BRPC/LAPC) with neoadjuvant chemotherapy and SBRT with or without surgical resection.

## 1. Introduction

Pancreatic adenocarcinoma (PDAC) is the eighth most common cancer and third leading cause of cancer-related deaths in the United States, accountable for over 48,000 deaths each year [1,2,3]. This number is expected to rise, making PDAC the second most common cause of cancer-related deaths by the year 2030 [4]. Roughly 50% of patients are diagnosed with localized disease [5]. The treatment of localized PDAC involves a combination of surgery and chemotherapy with or without radiation therapy. Despite aggressive multimodality therapy, outcomes remain suboptimal, with 5-year overall survival rates of less than 15%, even for patients with localized disease [2,6].

In the United States, most PDAC cases present in patients >55 years of age (median age of 70), with only 10% of patients diagnosed at <55 years of age [7,8]. Therefore, the optimal management of younger patients is largely unknown. Patients who develop pancreatic cancer at a young age are referred to as having Early-onset pancreatic cancer (EOPC). Various age cut-offs for EOPC (i.e., <40, <45, <50, <55 years) have been reported in the literature [9,10,11,12,13,14,15,16]. Previous studies have suggested that EOPC may behave more aggressively than Late-onset pancreatic cancer (LOPC), presenting at more advanced stages and having distinct molecular subtypes and mutations [17]. However, the literature on EOPC is limited, potentially due to late presentation or the lack of reportable case volume at single institutions.

Among the few existing reports on clinical outcomes and practice patterns in EOPC, it has been challenging to draw clear conclusions with respect to the optimal management of localized disease. Indeed, many of these studies have included heterogeneous patient populations, with the inclusion of both metastatic and localized disease, as well as highly variable treatment paradigms [9,14,15,16]. As such, there is a need for further investigation into the optimal treatment paradigm for EOPC across specific stages of the disease. Thus, we report on the clinical outcomes and toxicity of a cohort of patients with EOPC (age < 55 years) diagnosed and treated for borderline or locally advanced pancreatic cancer (BRPC/LAPC) with neoadjuvant chemotherapy and SBRT with or without surgical resection.

## 2. Materials and Methods

### 2.1. Study Design

This was a single-institution, retrospective study of patients <55 years old at the time of diagnosis who were treated between 2016 and 2021 for BRPC/LAPC with neoadjuvant chemotherapy and stereotactic body radiation therapy (SBRT), with or without surgical resection. Inclusion criteria were: (1) biopsy-confirmed diagnosis of PDAC; (2) BRPC/LAPC based on NCCN criteria; (3) age < 55; (4) neoadjuvant chemotherapy followed by SBRT; and (5) post-treatment follow-up with at least 1+ CT scan to evaluate for progression.

### 2.2. Definition of Outcomes

Clinical outcomes included overall survival (OS), local progression-free survival (LPFS), distant metastasis-free survival (DMFS) and progression-free survival (PFS). All events were measured from the completion of SBRT. OS was defined as the time to death or last known follow-up. LPFS was defined as the time to locoregional progression on imaging or last known negative imaging. DMFS was defined as the time to distant progression on imaging or last known negative imaging. PFS was defined as the time to any progression on imaging, death, or last known negative imaging. Radiation and chemotherapy-associated toxicity were graded per Common Terminology Criteria for Adverse Events (CTCAE). Surgical toxicity within the first 90 days was graded per Clavien–Dindo classification.

### 2.3. General Treatment Paradigm

Patients diagnosed with BRPC/LAPC were first treated with multi-agent neoadjuvant chemotherapy. At approximately three-month intervals, therapy response was assessed with pancreatic protocol computed tomography (CT) scans. The duration of chemotherapy was at the discretion of the treating medical oncologist and multi-disciplinary team. If disease was deemed stable or responsive after chemotherapy, patients were treated with five-fraction SBRT. The treatment details of SBRT have been described in a prior report [18]. Re-staging via CT imaging was performed after the completion of SBRT. BRPC patients with no medical contraindications and with imaging showing stable/responsive disease underwent surgical exploration with the goal of curative resection. A similar protocol was followed for those diagnosed with LAPC, unless the extent of vascular involvement on imaging was thought to preclude safe resection.

### 2.4. Statistical Analysis

Patient, treatment and disease characteristics such as age, sex, performance status, tumor location, disease extent, disease grade, cancer antigen 19-9 values, chemotherapy regimen/duration, SBRT dose/fractionation, and surgical information were recorded using descriptive statistics. Univariate analysis (UVA) was performed to identify associations with the aforementioned variable and clinical outcomes (OS, LPFS, DMFS, and PFS) (Figures S1–S4). Variables significant on UVA (*p* < 0.05) were analyzed with multivariable analysis (MVA) (Tables S1–S4). Kaplan–Meier analyses were performed for time to event outcomes, and the log-rank test was used to evaluate differences between groups. Cox regression was used as the type of regression analysis. Receiver operative characteristic (ROC) analysis was performed to identify optimal thresholds for CA 19-9 values. Statistical analysis was carried out with JMP version 15.0 (SAS institute, Cary, NC, USA).

## 3. Results

### 3.1. Patient, Disease and Treatment Characteristics

Table 1 displays the patient, treatment and disease characteristics of the cohort. From 2016 to 2021, 47 patients with EOPC (age < 55 years) were treated with neoadjuvant chemotherapy and SBRT, with or without surgical exploration. The median age was 50.4 years (range, 36.4–54.7), with a gender distribution of 31 males (66%) and 16 females (34%). Borderline resectable disease was seen in 21 (44.7%) patients, while LAPC was seen in 26 (55.3%) patients. The median baseline and post-chemotherapy CA 19-9 levels were 124.6 U/mL (range, 1.0–4361.0 U/mL) and 37.9 U/mL (1–326.4 U/mL), respectively. The median duration of induction chemotherapy was 4 months (range, 2.5–9 months), with most patients treated with modified FOLFIRINOX (mFFX) (39/47, 83%). Nearly all patients received SBRT to 33 Gy in five fractions (46/47, 97.9%), with one patient requiring a dose reduction to 30 Gy in five fractions to meet dose constraints. The majority of patients underwent surgical exploration (45/47), of which gross total resection was achieved in 33/47 (70.2%) patients. Two patients were not explored due to the extent of vascular involvement of the primary tumor on pre-operative imaging. Twelve patients were explored but not resected due to the extent of vascular involvement of the primary tumor (*n* = 4) or evidence of metastatic disease during exploration (*n* = 8). Of those who underwent surgical resection, 9/33 (27.3%) underwent vascular reconstruction, either arterial (1/33, 3%) or venous (8/33, 24.3%). Arterial reconstruction was conducted for the common hepatic artery to the aorta, and venous reconstruction was conducted for the portal vein to the superior mesenteric vein. Pathologic complete treatment response was only seen in 1/33 (3%) patients. Nodal positivity was seen in 26/33 (78.8%) patients. Lymphovascular invasion (LVI) and perineural invasion (PNI) were seen in 6/33 (18.2%) and 16/33 (48.5%) patients, respectively. Adjuvant chemotherapy was administered to 16/47 (34.0%) patients for a median duration of 2 months (range, 1–4 months).

### 3.2. Clinical Outcomes

The median follow-up for the entire cohort (*n* = 47) was 11.1 months (range, 1.9–39.9 months). At the time of the last follow-up, 19 patients had died, while the remaining 28 patients were either alive or lost to follow-up. The patterns of failure at the time of the last follow-up were as follows: 14 patients with distant metastases alone, 2 patients with local failure alone, and 16 patients with both distant and local disease. Of the 16 patients with both distant and local disease, 11 developed synchronous distant and local failure, and 5 developed distant failure first.

The median OS was 14.2 months. OS at 6 months, 1 year and 2 years were 93.0%, 64.4%, and 36.9%, respectively. The median OS for patients who received induction chemotherapy for ≥4 months was 16.5 months versus 10.1 months for patients who received <4 months of induction chemotherapy (log-rank *p* = 0.005). The median LPFS was 11.6 months. LPFS at 6 months, 1 year and 2 years were 88.3%, 45.4%, and 39.7%, respectively. The median LPFS for patients who received induction chemotherapy for ≥4 months was 10.1 months versus 4.9 months for patients who received <4 months of induction chemotherapy (log-rank *p* = 0.002).

The median DMFS was 8.9 months. DMFS at 6 months, 1 year and 2 years were 63.2%, 30.3%, and 22.7%, respectively. The median DMFS for patients who received induction chemotherapy for ≥4 months was 9.7 months versus 5.2 months for patients who received <4 months of induction chemotherapy (log-rank *p* = 0.014). The median DMFS was not reached in patients with the normalization of CA 19-9 to ≤34 U/mL versus 5.6 months in patients whom post-chemotherapy CA19-9 levels remained >34 U/mL (log-rank *p* = 0.003). The median PFS was 8.1 months. PFS at 6 months, 1 year and 2 years were 61.3%, 23.0%, and 17.3%, respectively. The median PFS for patients who received induction chemotherapy for ≥4 months was 9.7 months versus 5.2 months for patients who received <4 months of induction chemotherapy (log-rank *p* = 0.020). The median PFS was 11.3 months in patients with the normalization of CA 19-9 to ≤34 U/mL versus 5.6 months in patients whom post-chemotherapy CA19-9 levels remained >34 U/mL (log-rank *p* = 0.022).

### 3.3. Toxicity

Table 2 displays treatment related toxicities seen in our cohort. Chemotherapy grade ≥ 3-related toxicity was seen in seven (14.9%) patients, including neutropenic fever (3/7, 42.9%), failure to thrive (2/7, 28.6%), bacterial pharyngitis (1/7, 14.3%), and sepsis (1/7, 14.3%). There was one event of grade 5 toxicity that was potentially related to radiation. This patient had pancreatic adenocarcinoma of the head of the pancreas and suffered a fatal gastric bleed 5 months post-SBRT. She experienced sudden hematemesis and underwent an exploratory laparotomy with oversew of gastric ulcers followed by a subtotal gastrectomy with the creation of Roux-en-Y gastrojejunostomy. She passed away 6 days post-gastrectomy after suffering a syncopal episode in the setting of disseminated intravascular coagulation and hemorrhagic shock. Of note, there was significant abutment of the primary tumor to the distal stomach. The stomach constraint of V33 less than 1cc was met, although the maximum point dose to the stomach was 36.2 Gy in this patient. Surgical Clavien–Dindo grade 3b-related toxicity was seen in one patient (3%), with a bleeding pseudoaneurysm of the GDA 2 years after the resection of PDAC, which required two coil embolization procedures of the common hepatic artery.

## 4. Discussion

We report on a single-institution experience of patients with EOPC who were treated with neoadjuvant chemotherapy and SBRT with or without surgical resection. Longer induction chemotherapy duration (≥4 months) was associated with improved OS, LPFS, DMFS, and PFS, while the normalization of CA 19-9 (≤34 U/mL) after chemotherapy was associated with improved DMFS and PFS.

Patients diagnosed with PDAC at a young age are referred to as having EOPC. Various age cut-offs have been proposed, but EOPC is most commonly defined as a diagnosis before the age of 45–55 years [9,14,15,16]. In this study, we used an age-cut off of <55 years. Studies have shown that EOPC is treated more aggressively than its LOPC counterpart, likely due to fewer comorbidities and the better tolerance of aggressive systemic and local therapies in younger patients. However, despite aggressive therapies, many studies show that EOPC has similar outcomes as those for LOPC patients [16]. For example, Takeda et al. demonstrated that EOPC had a higher N stage and pathologic stage and lower rates of macroscopically curative resection while having a similar OS rate as LOPC [16]. It was thought that the poor tumor biology of EOPC was offset by the receipt of more aggressive therapy. Conversely, a few studies suggest that EOPC may have a better prognosis compared to LOPC, likely due to better performance status and tolerance of aggressive therapies [10,19,20,21]. Ntala et al. reported longer median survival for EOPC patients who underwent surgical resection than their LOPC counterparts [10]. This points to the question of whether tumor biology and external risk factors play a role in EOPC outcomes. Risk factors like diabetes, smoking, obesity, tobacco product use, chronic pancreatitis, alcohol abuse, and intrinsic factors, like hereditary pancreatitis and germline mutations, like BRCA mutations, are found in both populations, but EOPC patients may harbor a unique genetic profile [9,22].

The difference in mutational landscape and tumor biology between EOPC and LOPC may account for differences in treatment response [22]. A recent analysis suggests that KRAS mutations can predict local tumor response to neoadjuvant chemotherapy and SBRT [23]. A study by Ben-Aharon et al. demonstrated that patients ≤55 years of age had higher rates of SMAD4 mutations, which are seen in up to 50% of PDAC cases and associated with inferior outcomes [14,24,25,26,27]. Additionally, Tsang et al. showed the increased expression of FOXC2, a gene involved in the epithelial-to-mesenchymal transition, in EOPC. In the same study, they demonstrated increased rates of biallelic mutations of CDKN2A, a known tumor suppression involved in cell cycle regulation at the G1 checkpoint [15]. A better understanding of the mutational landscape of EOPC may help guide therapeutic decision-making with respect to the use of novel targeted agents, administration of radiation therapy, and formulation of an optimal multi-disciplinary treatment plan. Further investigation in this space is certainly warranted.

CA 19-9 is the most commonly used blood marker to track response to therapy among patients with pancreatic cancer. We show that the normalization of CA 19-9 to ≤34 U/mL post-chemotherapy and prior to SBRT led to improved DMFS and PFS, which is in agreement with the results from other studies [28,29,30,31]. Furthermore, these findings suggest that EOPC patients may derive the greatest benefit from titrating systemic therapy administration and duration to the normalization of CA19-9 before proceeding to aggressive local therapies, such as SBRT and surgical exploration. Those whose CA 19-9 levels do not normalize after chemotherapy are likely at risk of developing distant metastasis and may therefore benefit from the intensification of chemotherapy or transitioning to other therapeutic strategies. Furthermore, these patients may also derive less benefit from aggressive local therapy. This is in agreement with a study by Truty et al., who showed that the normalization (<37 U/mL) of CA 19-9 post-chemotherapy was associated with improved OS and recurrence-free survival in BRPC/LAPC treated with surgical resection following neoadjuvant chemotherapy and chemoradiation [28].

In this study, we also show that longer neoadjuvant chemotherapy duration led to improved outcomes (OS, LPFS, DMFS, and PFS) in our EOPC cohort. This is consistent with the findings from other studies, which included patients of all ages [32]. Furthermore, the rate of chemotherapy-related toxicity was low, with only 14.9% (*n* = 7) experiencing a G3 toxicity event. Given that EOPC patients likely have fewer comorbidities and the low rate of chemotherapy-related G3 toxicity, maximizing systemic therapy prior to transitioning to local therapies is imperative for improved outcomes. Indeed, more extended durations of chemotherapy, titrated to CA19-9 response, may be both warranted and feasible in this population.

As systemic therapy continues to improve, allowing patients to live longer, effective local control becomes increasingly important [33]. In this study, we demonstrate that local control was suboptimal despite the administration of neoadjuvant SBRT and the fact that most patients (70.2%) underwent surgical resection. Indeed, the median and 1-year LPFS rates were 11.6 months and 45.4%, respectively. This suboptimal locoregional control may be attributed in part to EOPC having different tumor biology that is resistant to traditional therapy and presenting at later stages with a greater tumor burden [15,22,24]. To overcome this, higher doses of SBRT may be warranted. Certainly, considerable data now supports dose escalation for pancreatic cancer, including recent prospective experience with ablative dosing [34,35,36,37]. In addition to dose escalation through external beam radiation therapy (EBRT), recent data have also supported novel approaches to dose escalation, including through intra-operative radiation therapy (IORT). Moreover, equally important is ensuring that target volume design is appropriate. While pre-operative radiation for pancreatic cancer originally focused on the vascular margin at risk, more recent data have demonstrated the importance of the elective targeting of at-risk tissue defined by the extra-pancreatic neural tract anatomy, often referred to as the “Triangle” volume [38,39,40]. Finally, given that younger patients are likely to have fewer comorbidities, aggressive surgical approaches may continue to be warranted. Indeed, nine (27.3%) patients underwent surgical resection with vascular reconstruction, with only one event of Clavien–Dindo grade 3b toxicity.

The limitations of this study should be mentioned, including its retrospective nature. The small sample size (*n* = 47) and single-institution nature also limit the generalizability of the findings. As such, these findings should be validated in a larger cohort. Additionally, this study is limited by a lack of a comparator group closer in age to the median age of diagnosis, and this analysis is limited by immortal time bias with respect to the importance of the duration of induction chemotherapy, as the case of some patients receiving <4 months of chemotherapy may have been due to the progression of disease rendering them candidates no longer for local therapy.

Furthermore, chemotherapy-related toxicity may have been underestimated because the cohort only included patients who completed their neoadjuvant chemotherapy course. The strengths of this study include its fairly homogenous practice patterns, including SBRT dose and fractionation (46/47 patients received 33 Gy in five fractions) and chemotherapy regimen (83% treated with mFFX alone). Despite the limitations, this study is the first, to our knowledge, to report on the outcomes of EOPC treated with neoadjuvant chemotherapy and SBRT.

## 5. Conclusions

In conclusion, multimodality therapy for EOPC was administered with low toxicity, but local control remains suboptimal. An induction chemotherapy duration ≥4 months and the normalization of CA 19-9 after chemotherapy were associated with improved outcomes, suggesting a role for extended durations of systemic therapy titrated to CA 19-9 response before transitioning to local therapy. The high rate of local failure and the low rate of grade 3+ treatment-related toxicity also suggest a role for intensifying local therapy in this population, such as via radiation dose escalation, the expansion of the radiation target volume by applying the Triangle volume, and more aggressive surgical techniques [38,39,40].

## Figures and Tables

**Table 1:** Patient, treatment, and disease characteristics.

	Total Cohort	Chemotherapy Duration < 4 mo	Chemotherapy Duration ≥ 4 mo
Characteristics	*N* (%) or Median (Range)	*N* (%) or Median (Range)	*N* (%) or Median (Range)
No. of patients	47	7	40
Age (years)	50.4 (36.4–54.7)	50.5 (39.5–53.9)	50.4 (36.4–54.7)
Sex			
Male	31 (66.0)	3 (42.9)	28 (70)
Female	16 (34.0)	4 (57.1)	12 (30)
ECOG			
0	25 (53.2)	3 (42.9)	22 (55)
1	22 (46.8)	4(57.1)	18 (45)
Location of primary tumor			
Head	27 (57.4)	5 (71.4)	22 (55)
Other	20 (42.6)	2 (28.6)	18 (45)
Disease extent			
Borderline resectable	21 (44.7)	6 (85.7)	15 (37.5)
Locally advanced	26 (55.3)	1 (14.3)	25 (62.5)
Baseline CA 19-9 (U/mL)	124.6 (1.0–4361.0)	441.7 (125.1–2185.1)	80.8 (1–4361)
Post-chemotherapy CA 19-9 (U/mL)	37.9 (1.0–3264.4)	57.7 (14.8–3264.4)	37.2 (1–1671.8)
Baseline total bilirubin (U/mL)	0.6 (0.2–23.1)	4.3 (0.4–23.1)	0.5 (0.2–13.2)
Induction chemotherapy			
Duration (months)	4 (2.5–9)	3 (2.5–3.5)	5 (4–9)
Regimen			
mFFX	39 (83.0)	6 (85.7)	33 (82.5)
GnP	3 (6.4)	0 (0)	3 (7.5)
mFFX and GnP	3 (6.4)	0 (0)	3 (7.5)
mFFX and GnP/cisplatin	1 (2.1)	0 (0)	1 (2.5)
GnP and gemcitabine	1 (2.1)	1 (14.3)	0 (0)
SBRT dose and fractionation			
33 Gy in 5 fractions	46 (97.9)	7 (100)	39 (97.5)
30 Gy in 5 fractions	1 (2.1)	0 (0)	1 (2.5)
Surgically resected	33 (70.2)	6 (85.7)	27 (67.5)
Whipple procedure	24 (72.7)	6 (85.7)	18 (45)
Distal pancreatectomy	8 (24.2)	0 (0)	8 (20)
Total pancreatectomy	1 (3.1)	0 (0)	1 (2.5)
Post-SBRT/surgery chemotherapy	16 (34.0)	1 (14.3)	15 (37.5)
Duration (months)	2 (1–4)	2 (NA)	2 (1–4)
Regimen			
mFFX	7 (43.7)	1 (NA)	6 (15)
GnP	4 (25.0)	0 (0)	4 (10)
FOLFIRI	1 (6.3)	0 (0)	1 (2.5)
Capecitabine	2 (12.4)	0 (0)	2 (5)
Gemcitabine	1 (6.3)	0 (0)	1 (2.5)
Unknown	1 (6.3)	0 (0)	1 (2.5)

Abbreviations: ECOG, Eastern Cooperative Oncology Group; CA 19-9, cancer antigen 19-9; mFFX, modified FOLFIRINOX; GnP, gemcitabine plus nab-paclitaxel; SBRT, stereotactic body radiation therapy.

**Table 2:** Treatment-related toxicity.

	*N* (%)	Description
Chemotherapy Grade ≥ 3	7 (14.9)	Neutropenic fever (*n* = 3), failure to thrive (*n* = 2), bacterial pharyngitis (*n* = 1), sepsis (*n* = 1)
Radiation Grade ≥ 3	1 (2.1)	Death due to upper gastrointestinal bleeding
Surgical Clavien–Dindo Grade 3b	1 (3.0)	Bleeding pseudoaneurysm of the GDA

## Data Availability

The data is stored internally at Johns Hopkins. If there is interest in obtaining the data, please contact the study team.

## Supplementary Materials

The following supporting information can be downloaded at: https://www.mdpi.com/article/10.3390/cancers18091418/s1, Table S1: Univariate analyses of overall survival; Table S2: Univariate analyses of local progression-free survival; Table S3: Univariate and multivariable analyses of distant metastasis-free survival; Table S4: Univariate and multivariable analyses of progression-free survival; Figure S1: Overall Survival (OS); Figure S2: Progression Free Survival (PFS); Figure S3: Local Progression Free Survival (LPFS); Figure S4: Distant Metastasis Free Survival (DMFS).

## Abbreviations

The following abbreviations are used in this manuscript:

PDAC	Pancreatic ductal adenocarcinoma
EOPC	Early-onset pancreatic cancer
LOPC	Late-onset pancreatic cancer
LAPC	Locally advanced pancreatic cancer
BRPC	Borderline resectable pancreatic cancer
SBRT	Stereotactic body radiation therapy
MFFX	Modified FOLFIRINOX
GnP	Gemcitabine plus nab-paclitaxel
ECOG	Eastern Cooperative Oncology Group
CA 19-9	Cancer antigen 19-9
CT	Computed tomography
OS	Overall survival
LPFS	Local progression-free survival
DMFS	Distant metastasis-free survival
PFS	Progression-free survival
