# Supplementary material for: Neoadjuvant Chemotherapy and Stereotactic Body Radiation Therapy in Patients with Early-Onset Pancreatic Cancer: Clinical Outcomes and Toxicity
Source: Cancers (Basel). 2026 Apr 29;18(9):1418. doi: 10.3390/cancers18091418 (PMC13162665; doi:10.3390/cancers18091418)
Supplement: Supplementary file 1 [file cancers-18-01418-s001.zip › cancers-4185331-supplementary.pdf]

Supplementary Material:

Additional Tables:

Table S1. Univariate analyses of overall survival.			
		UVA	
	HR	95% CI	P
Age (years)	1.08	0.93-1.28	0.316
Sex (male vs female)	0.47	0.18-1.24	0.126
ECOG (0 vs 1)	1.17	0.47-2.90	0.741
Disease extent (BRPC vs LAPC)	1.25	0.50-3.14	0.637
Tumor location (head vs other)	1.03	0.41-2.59	0.946
Induction CT duration (≥ 4 vs < 4 months)	0.24	0.08-0.70	**0.009**
Resected (yes vs no)	0.62	0.24-1.59	0.321
Baseline CA 19-9 (> 34 vs ≤ 34 U/ml)	0.46	0.15-1.41	0.174
Pre-SBRT CA 19-9 (> 34 vs ≤ 34 U/ml)	0.98	0.39-2.45	0.963
Baseline Total Bilirubin (mg/dl)	1.02	0.90-1.10	0.731
Post-SBRT/surgery chemotherapy (yes vs no)	0.46	0.17-1.30	0.143

Table S2. Univariate analyses of local progression-free survival.			
		UVA	
	HR	95% CI	P
Age (years)	1.01	0.89-1.17	0.887
Sex (male vs female)	1.50	0.43-5.26	0.530
ECOG (0 vs 1)	0.72	0.28-1.86	0.493
Disease extent (BRPC vs LAPC)	0.94	0.37-2.39	0.898
Tumor location (head vs other)	0.91	0.36-2.32	0.849
Induction CT duration (≥ 4 vs < 4 months)	0.16	0.04-0.58	**0.005**
Resected (yes vs no)	0.60	0.22-1.61	0.307
Baseline CA 19-9 (> 34 vs ≤ 34 U/ml)	1.09	0.30-4.00	0.898
Pre-SBRT CA 19-9 (> 34 vs ≤ 34 U/ml)	1.29	0.50-3.37	0.597
Baseline Total Bilirubin (mg/dl)	1.05	0.95-1.14	0.278
Post-SBRT/surgery chemotherapy (yes vs no)	0.63	0.23-1.69	0.358

Table S3. Univariate and multivariable analyses of distant metastasis-free survival.						
		**UVA**			MVA	
	**HR**	**95% CI**	P	HR	95% CI	P
Age (years)	1.01	0.99-1.12	0.771			
Sex (male vs female)	1.18	0.51-2.70	0.700			
ECOG (0 vs 1)	1.07	0.52-2.19	0.858			
Disease extent (BRPC vs LAPC)	1.01	0.49-2.07	0.983			
Tumor location (head vs other)	0.75	0.36-1.53	0.425			
Induction CT duration (≥ 4 vs < 4 months)	0.32	0.12-0.83	**0.019**	0.34	0.12-0.88	**0.027**
Baseline CA 19-9 (> 34 vs ≤ 34 U/ml)	3.45	0.96-12.34	0.057			
Pre-SBRT CA 19-9 (> 34 vs ≤ 34 U/ml)	3.48	1.46-8.26	**0.005**	3.29	1.38-7.84	**0.007**
Baseline Total Bilirubin (mg/dl)	0.99	0.88-1.08	0.798			

Table S4. Univariate and multivariable analyses of progression-free survival.						
		**UVA**			MVA	
	**HR**	**95% CI**	P	HR	95% CI	P
Age (years)	1.02	0.93-1.13	0.650			
Sex (male vs female)	1.12	0.51-2.45	0.785			
ECOG (0 vs 1)	1.06	0.53-2.12	0.862			

Disease extent (BRPC vs LAPC)	0.96	0.48-1.91	0.913			
Tumor location (head vs other)	0.66	0.33-1.32	0.239			
Induction CT duration (≥ 4 vs < 4 months)	0.34	0.13-0.88	**0.027**	0.35	0.13-0.92	**0.033**
Baseline CA 19-9 (> 34 vs ≤ 34 U/ml)	2.37	0.84-6.72	0.103			
Pre-SBRT CA 19-9 (> 34 vs ≤ 34 U/ml)	2.38	1.11-5.09	**0.026**	2.25	1.05-4.84	**0.038**
Baseline Total Bilirubin (mg/dl)	0.99	0.89-1.08	0.889			

Kaplan Meier Curves: Each of these curves have been stratified by Chemotherapy duration

1. **Figure S1.** Overall Survival (OS)

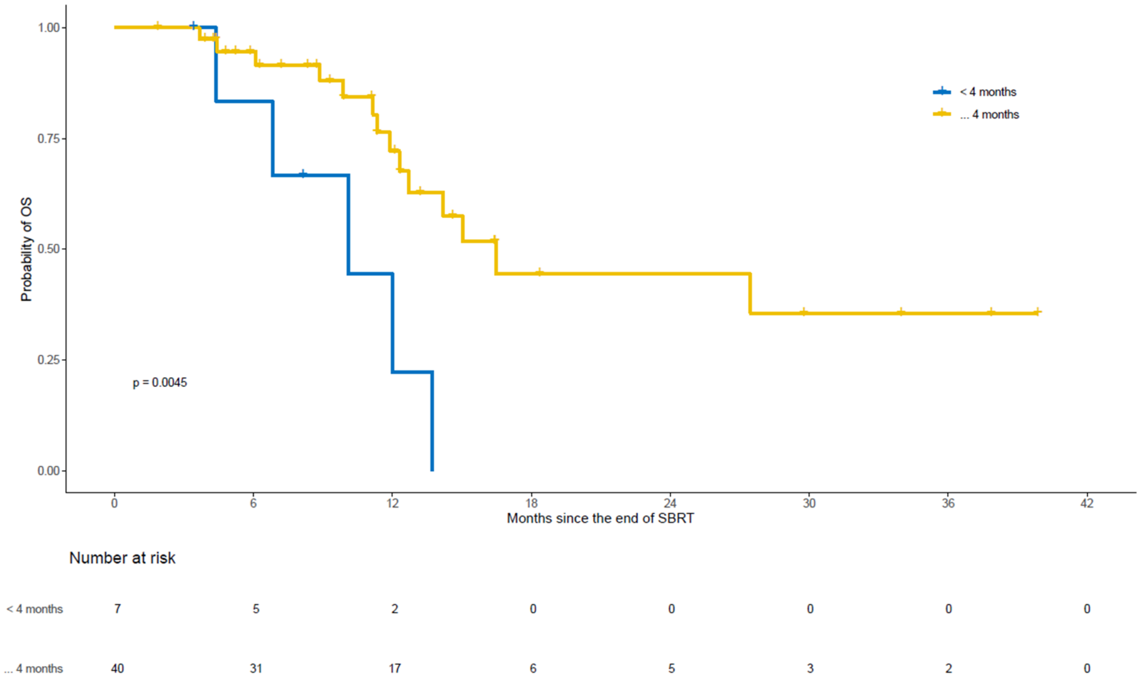

2. **Figure S2.** Progression Free Survival (PFS)

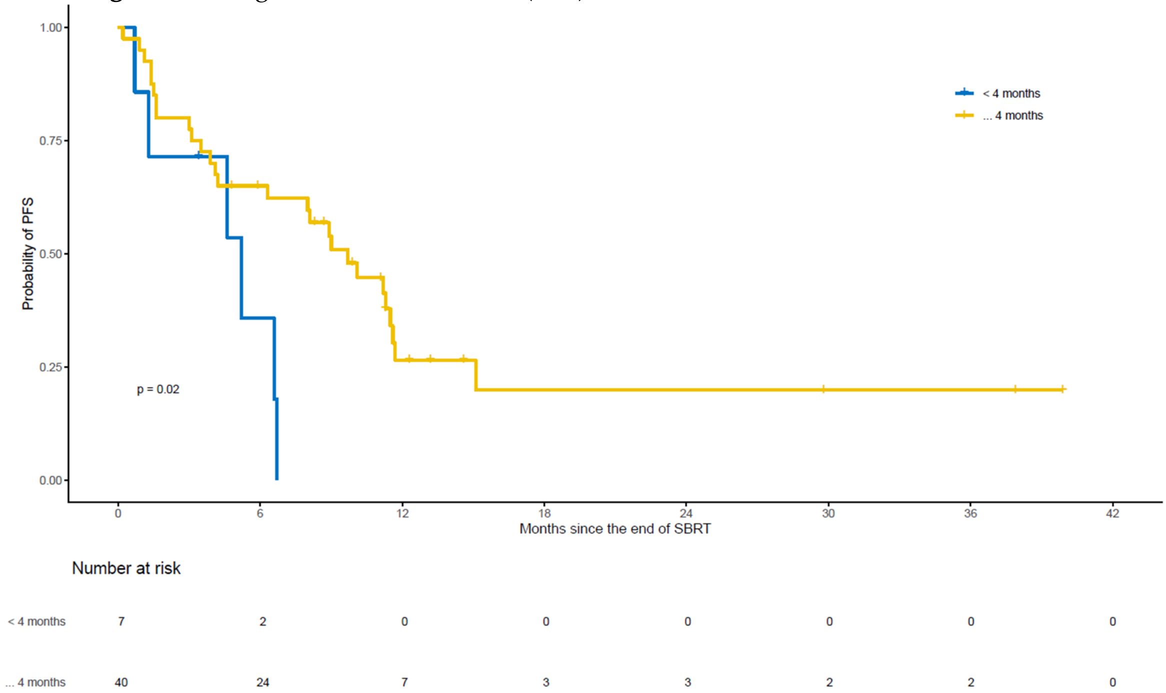

3. **Figure S3.** Local Progression Free Survival (LPFS)

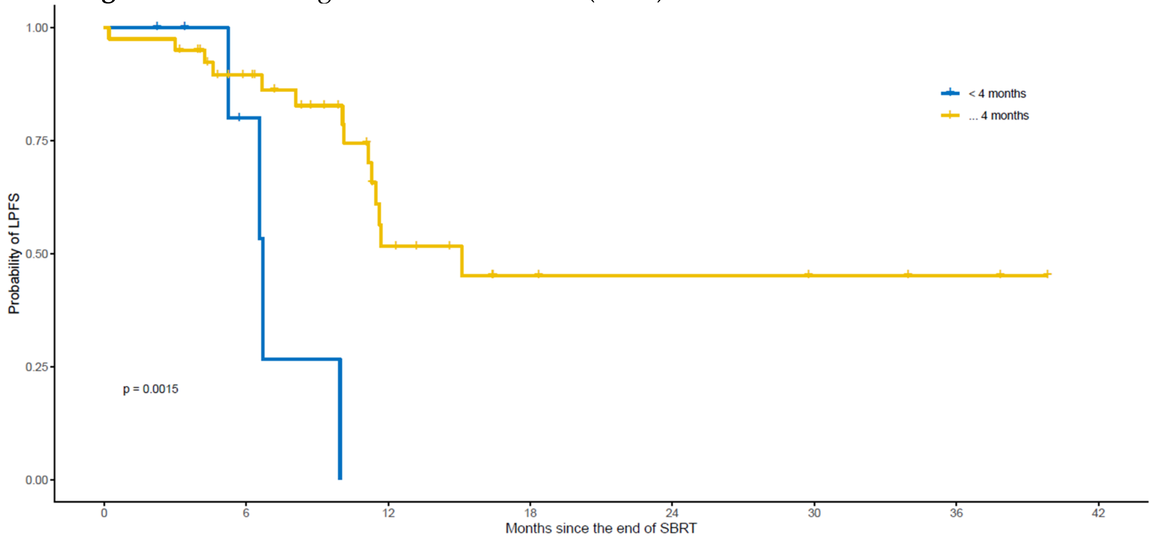

Number at risk

	0	6	12	18	24	30	36	42
< 4 months	7	3	0	0	0	0	0	0
... 4 months	40	29	11	5	4	3	2	0

4. **Figure S4.** Distant Metastasis Free Survival (DMFS)

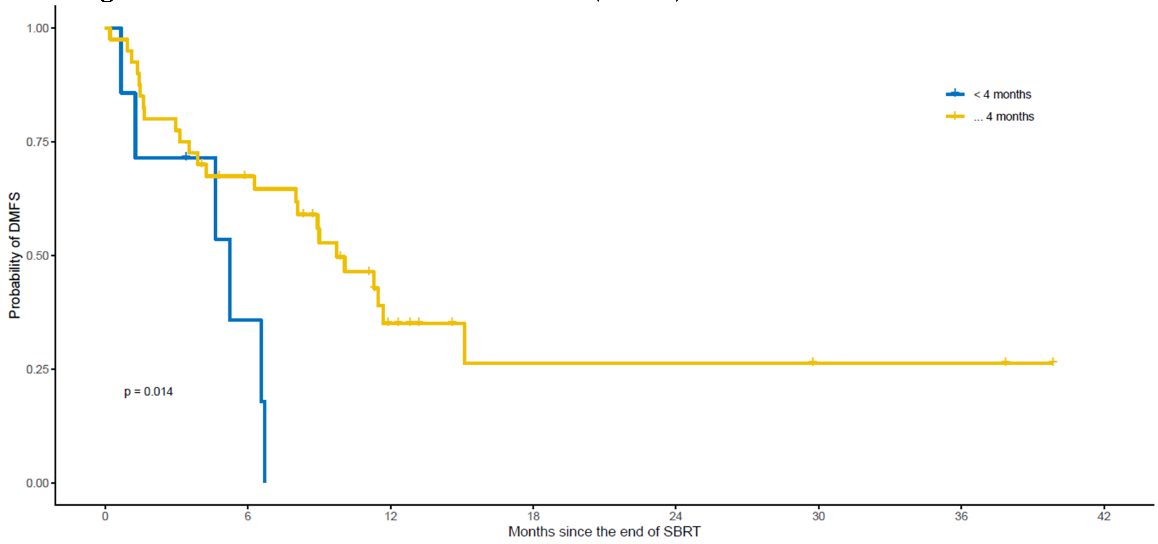

Number at risk

	0	6	12	18	24	30	36	42
< 4 months	7	2	0	0	0	0	0	0
... 4 months	40	24	8	3	3	2	2	0
